# Differential Expression of Serum Proinflammatory Cytokine TNF-α and Genetic Determinants of TNF-α, CYP2C19*17, miR-423 Genes and Their Effect on Coronary Artery Disease Predisposition and Progression

**DOI:** 10.3390/life13112142

**Published:** 2023-10-31

**Authors:** Rehab F. Almassabi, Rashid Mir, Jamsheed Javid, Faisel M. AbuDuhier, Reema Almotairi, Marwan H. Alhelali, Naseh Algehainy, Basim S. O. Alsaedi, Salem Owaid Albalawi, Imadeldin Elfaki

**Affiliations:** 1 Department of Biochemistry, Faculty of Science, University of Tabuk, Tabuk 71491, Saudi Arabia; rf-saif@ut.edu.sa; 2 Department of Medical Lab Technology, Prince Fahad Bin Sultan Chair for Biomedical Research, Faculty of Applied Medical Sciences, University of Tabuk, Tabuk 71491, Saudi Arabia; rashidmirtabuk@gmail.com (R.M.); jali@ut.edu.sa (J.J.); fabu-duhier@ut.edu.sa (F.M.A.); ralmotairi@ut.edu.sa (R.A.); nalgehainy@ut.edu.sa (N.A.); 3 Department of Statistics, University of Tabuk, Tabuk 47512, Saudi Arabia; malhilaly@ut.edu.sa (M.H.A.); balsaedi@ut.edu.sa (B.S.O.A.); 4 Department of Cardiology, King Fahd Specialist Hospital, Tabuk 71491, Saudi Arabia; sal-wabsy@moh.gov.sa.com

**Keywords:** coronary artery disease (CAD), tumor necrosis factor alpha (TNF-α), the Cytochrome p450s (CYP450), microR-423, Hardy–Weinberg disequilibrium (HWD), amplification refractory mutation system (ARMS)-PCR

## Abstract

Coronary artery disease (CAD) is the leading cause of death and hospitalization worldwide and represents a problem for public health systems everywhere. In Saudi Arabia, the prevalence of CAD is estimated to be 5.5%. Risk factors for CAD include older age, male gender, obesity, high blood pressure, smoking, diabetes, hyperlipidemia, and genetic factors. Reducing the risk factors in susceptible individuals will decrease the prevalence of CAD. Genome wide association studies have helped to reveal the association of many loci with diseases like CAD. In this study, we examined the link between single nucleotide variations (SNVs) of TNF-α-rs1800629 G>A, CYP2C19*17 (rs12248560) C>T, and miR-423 rs6505162 C>A and the expression of TNF-α with CAD. We used the mutation specific PCR, ARMS-PCR, and ELISA. The results showed that the A allele of the TNF-α rs1800629 G>A SNP is linked to CAD with odd ratio (OR) (95% CI) = 2.10, *p*-value = 0.0013. The T allele of the CYP2C19*17 (rs12248560) C>T is linked to CAD with OR (95% CI) = 2.02, *p*-value = 0.003. In addition, the A allele of the miR-423 rs6505162 C>A SNV is linked to CAD with OR (95% CI) = 1.49, *p*-value = 0.036. The ELISA results indicated that the TNF-α serum levels are significantly increased in CAD patients compared to healthy controls. We conclude the TNF-α rs1800629 G>A, CYP2C19*17, and miR-423 rs6505162 C>A are potential genetic loci for CAD in the Saudi population. These findings require further verification in future studies. After being verified, our results might be utilized in genetic testing to identify individuals that are susceptible to CAD and, therefore, for whom reducing modifiable risk factors (e.g., poor diet, diabetes, obesity, and smoking) would result in prevention or delay of CAD.

## 1. Introduction

Coronary artery disease (CAD) represents a health problem all over the world, including in Saudi Arabia [1]. CAD is defined as an inadequate amount of blood supply to the myocardium by the coronary arteries and is the most common risk factor for cardiac disorders [2]. Myocardial ischemia or a lack of blood flow to the heart muscles can cause myocardial infarction or cardiac death. [2]. Atherosclerosis is the primary cause of CAD [3]. Chronic inflammation from CAD causes malfunction in the vascular endothelial cells, which, in turn, causes a buildup of lipoproteins in the intima of large and medium-sized arteries, an increase in connective tissues, the activation of thrombocytes, and the recruitment of immune cells [4]. The recruited immune cells secrete pro-inflammatory cytokines [4,5]. Risk factors for atherosclerotic cardiovascular disease include hyperlipidemia, metabolic syndrome, sex, chronic inflammatory disorders, chronic kidney disease, diabetes, obesity, smoking, and genetic factors [6].

Activated macrophages, T cells, and natural killer cells create tumor necrosis factor alpha (TNF-α), a pro-inflammatory cytokine homodimer protein, which has 157 amino acid residues [7]. It regulates inflammatory responses, and it plays roles in inflammatory disorders and autoimmune diseases [7]. There are soluble and transmembrane forms of TNF-α. It is first synthesized as the precursor form, transmembrane TNF-α, which is then converted to soluble TNF-α in a reaction catalyzed by the metalloproteinase TNF-α-converting enzyme (TACE) [7]. TNF-α plays important roles in the induction and progression of CAD [8]. It has been reported that high levels of TNF-α are associated with increased risk of cardiovascular disease and decreased risk of cancer [9]. High levels of TNF-α also contribute to the development of atherosclerosis, its progression, and the rupture of atherosclerotic plaque [10].

Cytochrome p450s (CYP450) is a superfamily of enzymes catalyzing the biotransformation of endogenous and exogenous substrates [10,11]. These enzymes play a crucial role in the metabolism of xenobiotics including drugs and endogenous substrates such as cholesterol, steroid hormones, and fatty acids [10,12]. It has been reported that members of the CYP450 family catalyze more than 90% of the oxidation–reduction reactions of the metabolism of all chemicals [13]. They are found in liver and tissues such as lungs, intestines, brain, gonads, adrenal glands, and kidneys [14,15]. Single nucleotide variations (SNVs) in CYP450s genes are associated with dysfunctional CYP450 enzyme, resulting in various illnesses [16].

**Figure 1:**
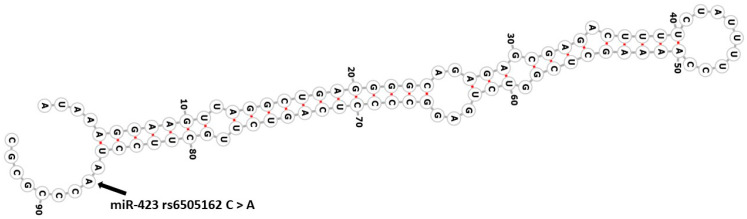
Structure of the miR-423 rs6505162 C>A. Structure of the miR-423 indicating the site of single nucleotide variation, rs6505162 C>A. This figure was prepared using the RNAfold web server, http://rna.tbi.univie.ac.at/cgi-bin/RNAWebSuite/RNAfold.cgi (accessed on 20 September 2023).

MicroRNAs are short, non-coding RNA molecules; they play very important roles in the regulation of their target gene expression [17]. MiRNAs bind and interact with the 3′ untranslated region (3′ UTR) of their target mRNAs [17]. This binding causes suppression of translation and mRNA degradation of the target genes [17]. MicroRNAs regulate the genes involved in important physiological processes such as metabolism, cell division, differentiation, and apoptosis [18,19]. MiR-423-5p is found on chromosome 17 [20]; it regulates insulin receptor substrates 2 and Phosphoinositide-3-Kinase Regulatory Subunit 3 PIK3R3 gene and, therefore, may be involved glucose and lipid metabolism [21]. In the present study, we evaluated the association of TNF-α rs1800629 G>A, CYP2C19*17 (rs12248560) C>T, and miR-423 rs6505162 C>A (Figure 1) with CAD in the Saudi population.

## 2. Methodology

### 2.1. Study Population

This case-control study was conducted on clinically confirmed cases of CAD. All subjects were recruited from King Fahd Special hospital, Tabuk, Saudi Arabia. The University of Tabuk’s ethical committee granted approval for this research project (UT-91-23-2020). All CAD patients and healthy control individuals provided their informed consent prior to the collection of blood samples.

#### 2.1.1. Patient Selection Criteria

Criteria for Inclusion of Subjects in this Study

We chose cases at the King Fahad Specialist Hospital in Saudi Arabia undergoing elective angiography for the diagnosis of stable angina. In addition, tests for exercise stress, myocardial perfusion imaging, ambulatory electrocardiography, Holter monitoring, X-rays, echocardiograms for the chest, cardiac computed tomography, and multigated acquisition scans (MUGA) were performed.

According to the results of the coronary angiography, subjects were split into three groups: those with substantial CAD, those with ischemic heart disease (stenosis 50%), and those without ischemic heart disease (no stenosis or stenosis 50%). This study excluded subjects with cancer, diabetes, or other chronic diseases.

#### 2.1.2. Healthy Controls

Subjects who regularly checked in at King Fahd Specialist Hospital served as healthy controls. The healthy controls appeared to be in good health and had no history of heart disease or any other chronic illnesses. For the health controls, blood biochemistry tests were also carried out.

#### 2.1.3. Genomic DNA Extraction

Each subject’s genomic DNA was purified using DNeasy Blood K (Qiagen, Hilden, Germany) in accordance with the manufacturer’s instructions. The Nanodrop was used to measure the amount of DNA, and agarose gel electrophoresis was used to evaluate the integrity of the DNA. DNA was then stored at 25 °C until genotyping was done.

#### 2.1.4. Genotyping of TNF-α rs1800629 G>A, miR-423rs6505162C>A and CYP2C19*17-rs12248560 *C>T* was Determined by ARMS-PCR Using Tetra-Primers

A PCR reaction was carried out in a reaction volume of 12 µL comprising template DNA (50 ng), FO—0.12 µL, RO—0.12 µL, FI—0.12 µL, RI—0.12 µL (25 pmol of each primer), and 6 µL from Green PCR Master Mix (2×) Cat M712C from (Promega, Madison, WI USA). Nuclease-free ddH2O was added to adjust the volume to a final value of 12 µL. Primers were previously used for TNF-α rs1800629 G>A, miR-423rs6505162C>A and CYP2C19*17-rs12248560 *C>T* (Table 1). The thermocycling conditions were 95 °C for 10 min, then 30 cycles of 95 °C for 30 s, 60 °C for genotyping TNF-α rs1800629 G>A, 62 °C for miR-423 rs6505162 C>A, 56 °C for CYP2C19*17-rs12248560 C>T for 35 s, 72 °C for 40 s, and finally, 72 °C for 10 min.

**Figure 2:**
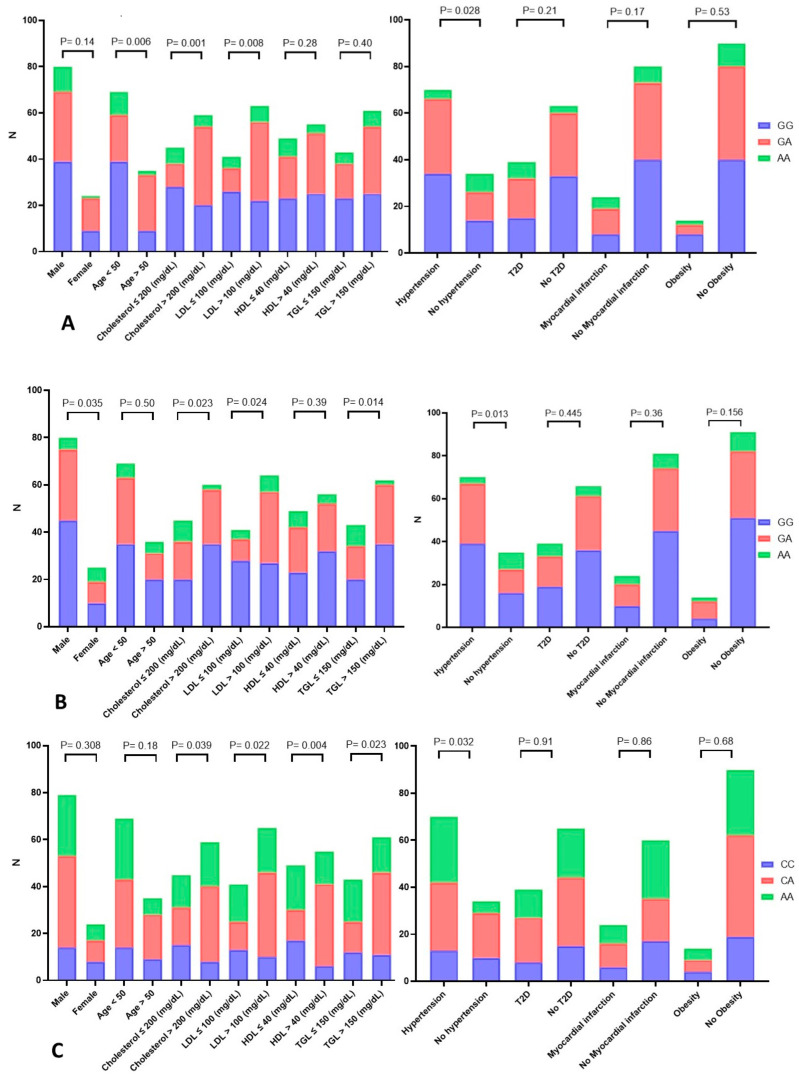
The genotypes of the single nucleotide variations (SNVs) and patient characteristics. (**A**) TNF-α rs1800629 G>A, (**B**) CYP2C19*17—rs12248560 C>T, and (**C**) miR-423 rs6505162 C>A. The *p*-values > 0.05 were considered significant.

After amplification, the PCR products were separated by gel electrophoresis on a 2% agarose gel and visualized with a UV transilluminator from Bio-Rad in Hercules, CA, USA, using syber safe stain. With the help of the amplification-refractory mutation system PCR (ARMS-PCR), the three examined genes were genotyped. Finally, agarose gel electrophoresis (2.5%) was used to separate the PCR products created through PCR amplification, and the appropriate bands were detected for the wild type or mutant allele, as shown in Figure 2. To flank the exon/intron of the TNF-α gene, primers Fo and Ro were created, which produced a 323 bp band. This band allowed us to determine whether the DNA was intact. Additionally, a 224 bp band corresponding to the G allele was produced when the Fo and RI primers were employed. As an alternative, the 154 bp band of the A allele was generated by primers FI and RO. The exterior region of the CYP2C19*17 gene was amplified using the Fo and Ro external primers, yielding a 462 bp band that served as a control for assessing DNA integrity. The FO and RI primers also produced a band of 292 bp corresponding to the C allele, while the FI and RO primers produced a 227 bp band corresponding to the T allele. The external region of the IL-8 gene was amplified using the FO and RO external primers, yielding a 336 bp band that served as a control for assessing DNA integrity. In addition, the FO and RI primers generated a band of 160 bp that matched the A allele, whereas the FI and RO primers generated a band of 228 bp that matched the A allele.

#### 2.1.5. Statistical Analysis

Students’ two-sample *t*-tests or one-way analyses of variance were used to distinguish between the various groups for continuous variables. The two tests were also used for categorical variables like departures from Hardy-Weinberg equilibrium. Additionally, a Chi-square test was utilized to assess the genotypic and allelic frequencies between the TNF-, miR-423, and CYP2C19*17 gene groups.

By estimating the odds ratios, risk ratios, and risk differences with 95% confidence intervals, possible associations between the TNF-rs1800629 G>A, miR-423 rs6505162C>A, and CYP2C19*17-rs12248560 C>T genotypes with CAD cases were evaluated. A *p*-value of 0.05 or less was considered significant. Software from Graph Pad Prism 8.4 and SPSS (version x86-64) (Chicago, IL, USA and San Diego, CA, USA, respectively), were used to conduct the analyses.

### 2.2. Estimation of Serum Expression of TNF-α

The TNF-α serum levels were assayed using kits from Diaclone, SAS, Besançon cedex, France, according to the manufacturer’s instructions. Less than 10% of both intra- and inter-assay variability was observed. The human TNF-α ELISA kit with catalogue number 950.090.096 was used to estimate TNF-α, and the results were expressed as pg/mL.

## 3. Results

### 3.1. Demographic Features of CAD Patients

The demographic patterns seen in 104 consecutive CAD patients are summarized in Table 2. Clinical information was gathered for all 104 CAD patients from all CAD profiles; however, patients were divided based on age into two groups, i.e., >50 years (*n* = 35, 33.65%) and 50 years (*n* = 69, 66.34%), as shown in Table 2. Out of 105 CAD cases, 24 were females and 80 were males.

### 3.2. Lipid Biomarkers

According to the results shown in Table 2, 61% of participants had TGL levels above 150 mg/dL, 60.57% of participants had LDL levels above 100 mg/dL, 52.88% of participants had HDL levels above 40 mg/dL, and 59% of participants had cholesterol levels above 200 mg/dL. In 104 CAD instances, there were 104 cases of hypertension, 37.5% T2D (+), 13.46% of obesity, and 24% of MI. The clinical features of the healthy controls are shown Table 3.

### 3.3. Hardy–Weinberg Equilibrium for Genotype Distributions and Allele Frequencies

The genotype distributions and allele frequencies of the SNPs for the four genes showed little or no variation. The control group had TNF-α-rs1800629 G>A, CYP2C19*17-rs12248560 C>T, and miR-423 rs6505162 C>A. As a result, the genotyping findings from 10% of the samples randomly selected from the healthy control group were evaluated. These results showed an accuracy of more than 99%. *p*-values > 0.05 were considered significant.

### 3.4. Frequency, Distribution, and Association of Genotypes and Alleles of TNF-α G>A, CYP2C19*17—C>T, and miR-423 C>A between CAD Patient and Controls

At the time of analysis, CYP2C19*17—rs12248560 C>T, miR-423 rs6505162 C>A, and TNF-α-rs1800629 G>A SNP were studied in 105 CAD cases and 114 controls.

#### 3.4.1. Statistical Analysis to Estimate the Association of TNF-α rs1800629 G>A Genotypes with CAD Patient Susceptibility

In CAD patients and controls, the TNF-α rs1800629 G>A genotype frequency was GG (46.15%), GA (42.30%), and AA (11.53%), whereas in controls, it was GG (67.30%), GA (27.88%), and AA (4.80%) (Table 4). The frequency and distribution of TNF-α rs1800629 G> genotypes between CAD patients and controls were statistically significant (*p* = 0.006). Furthermore, it was found that CAD patients had a higher frequency of the A allele than healthy individuals (0.33 vs. 0.19; Table 4). Additionally, it was discovered that CAD patients had a higher frequency of the A allele (0.33 vs. 0.19) than healthy people (Table 4).

#### 3.4.2. Statistical Analysis to Estimate the Association of the CYP2C19*17—rs12248560 C>T Genotypes with the CAD Patient Susceptibility

In CAD patients and controls, the frequency of the CYP2C19*17—rs12248560 C>T genotype was CC (52.88%), CT (37.5%), and TT (10.47%), respectively (Table 4), and in controls, it was CC (70.19%), CT (25.96%), and TT (3.84%), respectively. The frequency and distribution of CYP2C19*17—rs12248560 C>T between CAD patients and controls were statistically significant (*p* = 0.018). Additionally, it was observed that the T allele was more common in CAD patients (0.29 vs. 0.16) than in healthy people.

#### 3.4.3. Statistical Analysis to Estimate the Association of the miR-423 rs6505162 C>A Genotypes with CAD Patient Susceptibility

In contrast to controls, who were more likely to have frequencies of CC (28.07%), CA (54.38%), and AA (17.34%), respectively, CAD cases had miR-423 rs6505162 C>A frequencies of CC (22.11%), CA (46.15%), and AA (31.73%) (Table 4). In CAD patients and healthy controls, there was a statistically significant difference in the miR-423 rs6505162 C>A genotypes (*p* = 0.049). Additionally, it was found that CAD patients had a higher frequency of the A allele (0.54 vs. 0.45) than healthy controls.

### 3.5. Logistic Regression to Estimate the Association of TNF-α rs1800629 G>A Genotypes with CAD Patient Susceptibility

The results of the codominant model indicated a probable link between the TNF-α—GA genotype and higher CAD susceptibility, with OR = 2.21, (95%) CI = (1.219 to 4.0138), RR = 1.49 (1.084 to 2.055), and *p* = 0009 (Table 5). However, the TNF-α—AA genotype had a high OR of 3.12 (95%), CI = (1.1580 to 10.578), and RR = 2.1 (0.9514–0.2761) association with CAD susceptibility. In the codominant inheritance model, *p* = 00.02. The TNF-α—GG and (GA + AA) genotypes are strongly associated with an increased risk of developing coronary artery disease (OR = 2.40, 95% CI (1.368 to 4.215), RR = 1.57 (1.1583 to 2.1289), and *p* = 0.002), according to the dominant inheritance model (Table 5). The effects of the TNF-α—(GG + GA) and TNF-α—(AA) genotypes on CAD susceptibility in the recessive inheritance model were not found to be statistically significant (OR = 5.60, RR = 1.76, and *p* = 0.085; Table 5). The TNF-α—A allele is substantially related with CAD susceptibility in allelic comparison, with an OR of 2.10, (95% CI) (1.33 to 3.31), RR1.50 (1.145 to 1.965), and *p*-value = 0.001. The TNF-α—GA and T TNF-α—GG + AA genotypes have a statistically significant impact on CAD propensity in the over dominant inheritance model, with OR = 1.89, (95%) CI (1.063 to 3.383), RR = 1.30 (1.15 to 1.92), and *p*-value = 0.030 (Table 5).

### 3.6. Logistic Regression to Estimate the Association of CYP2C19*17 (rs12248560) C>T Genotypes with CAD Patient Susceptibility

To determine the relationship between the CYP2C19*17 C>T genotype and risk of CAD, a multivariate analysis based on logistic regression was performed. Odds ratios (OD) and risk ratios (RR) with 95% confidence intervals (CI) were obtained for each group. (Table 6) Our findings showed that the CYP2C19*17—TT genotype was highly related with higher CAD susceptibility in the codominant inheritance model, with OR 1.91 (95%), CI = (1.0493 to 3.5028), and RR = 1.39(1.0056 to 1.9326), *p* < 0.030. (Table 6). In the codominant inheritance model, the heterozygous CYP2C19*17—CT genotype was linked to CAD susceptibility, with OR 3.65 (95% CI = 1.1030 to 12.078) and RR = 2.13 (0.9117 to 5.0167) (*p* = 0.034. (Table 6) The CYP2C19*17—CC vs (CT + TT) genotypes were found to be significantly associated in the dominant inheritance model, with OR 2.14 (95%), CI = (1.2124 to 3.7799), and RR = 1.49 (1.2124 to 3.7799), *p*-value = 0.008. The CYP2C19*17-TT vs. CYP2C19*17-(CC + CT) genotypes were highly related to higher CAD susceptibility according to a recessive inheritance model, with OR 2.92 (95%), CI = (0.9003 to 9.5067), and RR = 1.93 (0.8260 to 4.5236), *p* < 0.074. (Table 6) No connection with CAD susceptibility was observed in allelic comparison, with OR 2.02 (95% CI) (1.2650 to 3.2371), RR 1.47 (1.1106 to 1.9554), and *p*-value = 0.003 for both (Table 6).

### 3.7. Logistic Regression to Estimate the Association of the miR-423 rs6505162 C>A Genotypes with CAD Patient Susceptibility

Our results showed that the miRNA-423—CC genotype vs. CA genotypes was not linked with CAD susceptibility (codominant model) with OR 1.07, 95% CI = (0.5595 to 2.0737), RR = 1.03 (0.7818 to 1.3630), and *p* = 0.82. In contrast, the miRNA-423—AA genotype was linked to CAD susceptibility, with an OR of 2.29 (95% CI: 1.0611–4.9667) and RR of 1.54 (1.0611–4.9667), *p* = 0.034, in the model of codominant inheritance. (Table 7) Additionally, the miR-423-CC genotype was not related to an increased risk of developing CAD in the dominant inheritance model when compared to the miR-423-(CA + AA) genotype, with OR 1.37, 95% CI = (0.7411 to 2.5485), RR = 1.15 (0.8819 to 1.5167), and *p* = 0.31 (Table 7). Recessive inheritance model research found a high connection between the miR-423-AA and miR-423 (CC + CA) genotypes with enhanced CAD susceptibility, with OR 2.18 (95%) CI = (1.1574 to 4.1230), and RR = 1.50 (1.0424 to 2.1865), respectively. In addition, the A allele was connected to CAD susceptibility in allelic comparison, as shown in the table, with OR 1.49, 95% CI = 1.0268 to 2.1858, RR 1.21 (1.0116 to 1.4541), and *p*-value = 0.036 (Table 7).

### 3.8. Statistical Analysis to Estimate the Association between the TNF-α rs1800629 G>A Genotypes and Demographic and Clinical Variables of CAD Patients

Our findings showed a significant relationship (*p*-value = 0.022) between the age of CAD patients and the TNF-α rs1800629 G>A genotypes (Figure 2A). We also found a strong correlation (*p*-value = 0.001) between TNF-α rs1800629 G>A genotypes and cholesterol (mg/dL) levels in the blood of CAD patients. Additionally, the findings demonstrated a strong correlation between TNF-α rs1800629 G>A genotypes and LDL-C (mg/dL) of CAD patients with *p* = 0.0080). Moreover, our findings showed a substantial association between the TNF-α rs1800629 G>A genotype and hypertension (*p*-value = 0.002) in CAD patients. In CAD patients, a strong connection between TNF-α rs1800629 G>A genotypes and diabetes was found (*p*-value = 0.028).

### 3.9. Statistical Analysis to Estimate the Association between the CYP2C19*17 (rs12248560) C>T Genotypes and Demographic and Clinical Variables of CAD Patients

According to our findings, there was a significant correlation between the gender of the CAD patients and their CYP2C19*17 C>T genotypes (*p*-value = 0.022; Figure 2B). However, it was found that male CAD patients had a higher incidence of heterozygosity than female CAD patients. A significant correlation (*p*-value = 0.023) between the genotypes of CYP2C19*17 (rs12248560) C>T and blood cholesterol levels in CAD patients was found in our statistical analysis of the data. Additionally, the results demonstrated a significant relationship (*p =* 0.024) between coronary artery disease patient LDL-C (mg/dL) and CYP2C19*17 C>T genotypes. CYP2C19*17 C>T genotypes and the triglyceride levels (mg/dL) of coronary artery disease patients were shown to be strongly correlated (*p*-value = 0.014). Our findings showed a significant relationship (*p*-value = 0.013) between the genotypes of CYP2C19*17 C>T and hypertension (Figure 2B).

### 3.10. Statistical Analysis to Estimate the Association between miR-423 rs6505162 C>A Genotypes and Demographic and Clinical Variables of CAD Patients

A significant connection (*p*-value = 0.039) was found in our statistical study of the relationship between the genotypes of miR-423 rs6505162 C>A and the levels of cholesterol (mg/dL) in the blood of individuals with CAD (Figure 2C). Additionally, the results demonstrated a strong correlation between the miR-423 rs6505162 C>A genotypes and LDL-C (mg/dL) in individuals with CAD (*p*-value = 0.020). Our findings demonstrated a substantial significant correlation (*p*-value = 0.004) between the miR-423 rs6505162 C>A genotypes and HDL-C (mg/dL) of individuals with CAD. The MiR-423 rs6505162 C>A genotypes and triglycerides levels (mg/dL) of coronary artery disease patients showed a significant correlation (*p*-value = 0.023). Our findings showed a significant association between the miR-423 rs6505162 C>A genotype and hypertension in patients with CAD (*p*-value = 0.032).

### 3.11. Estimation of the TNA Serum Levels between Cases and Controls Using ELISA

**Figure 3:**
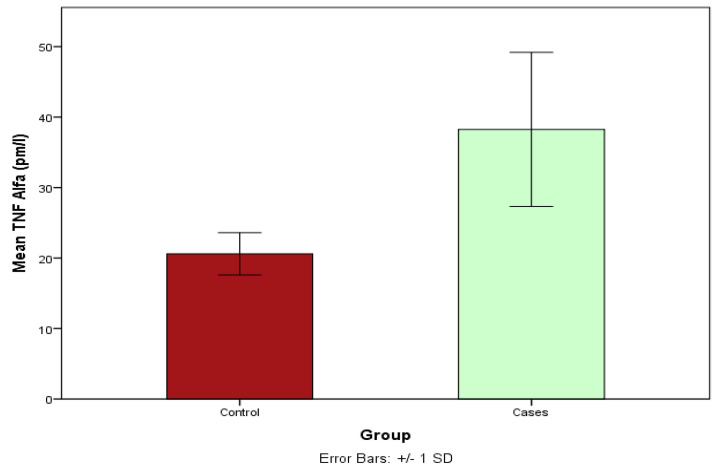
Serum TNF-α in cases and controls. Tumor Necrosis Factor Alpha (TNF-α) serum concentration (pg/mL) in CAD cases and healthy controls showing significant differences. Control vs. cases, 20.60 ± 2.99 vs. 38.25 ± 10.93, *p*-value ≤ 0.001, T = −11.14.

The TNF-α levels were compared between cases and controls using an unpaired *t* test. The result showed that the TNF alpha levels were statistically significantly higher in CAD cases compared to controls (*p* < 0.001); see Figure 3.

## 4. Discussion

It has been reported that in the year 2020, there were 19.1 million deaths worldwide due to CAD [1], with a prevalence of about 5.5% in Saudi Arabia [25]. Genome wide association studies (GWAs) have uncovered the linkage of specific loci with diseases including cancers, T2D, and cardiovascular disease [26,27]. Our results showed there was a significant difference in the TNF-α—308 (rs1800629) G>A genotype distribution between CAD cases and healthy controls (Table 4). We also found that the GA genotype and the A allele of TNF-α—308 G>A (rs1800629) are associated with CAD (Table 5). Our results showed that there was an association between the TNF-α rs1800629 G>A genotypes, hypercholesterolemia, and hypertension (Figure 2A). This result is consistent with studies reporting that TNF-α is involved in hypertension [28,29]. Hypertension is a risk factor for cardiovascular disease [30]. The A allele of the TNF-α-308 G>A (rs1800629) increases the expression of the TNF-α protein [31]. In carriers of the GA genotype, there is more TNF-α messenger RNA and protein than in the carriers of the GG genotype [31]. This result is partially consistent with previous studies reporting that elevated TNF-α is associated with cardiovascular disease, inflammation, atherosclerosis, obesity, insulin resistance, and increased blood pressure [2,31,32].

Atherosclerosis, the main cause of CAD, is a chronic inflammation of the wall of the arteries developed by the cells of the immune system and cytokines such as TNF-α in the arterial wall. It has been observed that mice that are deficient in TNF-α show decreased atherosclerotic plaque size [9]. Increased plasma levels of TNF-α after myocardial infarction (MI) is a risk factor for recurrent MI [9]. In addition, the inflammation induced by pro-inflammatory (e.g., TNF-α) responses is associated with the development of atherosclerosis [2]. TNF-α is vital for the inflammatory response and activation of downstream targets; these targets are activated when TNF-α binds to its receptors, i.e., TNFR1 (p55) and TNFR2 (p75) [2]. The exact role of TNF-α in the induction of atherosclerosis remains to be elucidated in future studies. Anti-TNF-α treatment has been suggested as a therapeutic strategy for CVD [2,33].

TNF-α causes disruption of endothelial cell connections and increases the permeability of blood vessels [34,35]. This disruption results in the invasion of immune cells and causes different diseases of blood vessels such as atherosclerosis [34,35]. Elevated levels of TNF-α activate the endothelial cells to produce adhesion molecules, which, in turn, control the attachment of immune cells to tissue and the induction of diapedesis [2]. TNF-α increases NF-κB-mediated reactive oxygen species (ROS) production and LDL transcytosis while reducing the production of NO levels and decreasing nitric oxide production in vasculature, which results in endothelial dysfunction and the initiation of atherogenesis [2,36]. Elevated TNF-α levels also stimulate the formation of phagocytic scavenger receptor, foam cells, and the proliferation of endothelial smooth muscle cells, which contribute to atherosclerosis [2,37]. Our genotyping results, i.e., GA and A allele of TNF-α—308 G>A (rs1800629), are associated with CAD (Table 4 and Table 5). This is in agreement with the ELISA result, which showed that the serum TNF-α concentration is elevated in CDA cases compared to healthy controls (Figure 3). This is probably because the A allele of the TNF-α-308 G>A (rs1800629) increases the expression of the of the TNF-α protein [31]. It has been reported that serum cytokines are involved in the development of atherosclerosis, and the serum levels of these cytokines (such as TNF-α) are associated with CAD [38]. Elevated levels of TNF-α play important roles in the induction of atherosclerosis [34,35].

Our results demonstrated that the CYP2C19*17 (rs12248560) C>T genotype distribution is significantly different between CDA cases and controls (Table 4). The CYP2C19*17 (rs12248560) CT genotype and T allele are linked to CAD (Table 6). Our results also showed that the CYP2C19*17 (rs12248560) C>T genotype distribution was significantly different in CAD cases with hypercholesterolemia and hypertriglyceridemia and cases with normal total cholesterol and normal triglyceride (TGL) (Figure 2B). In addition, there was significant distribution in the CYP2C19*17 (rs12248560) C>T genotype in cases with hypertension and normal blood pressure (Figure 2B). The CYP2C19*17 (rs12248560) C>T SNP increases the transcription of CYP2C19 [39]. Our results are in line with the study by Gaio et al. [40] that reported an association between CYP2C19 gene variations and susceptibility to metabolic syndrome in a South Portuguese population [40]. Our results are also consistent with those of Bai et al., who reported an association of CYP2C19 gene variations with lipid metabolism in patients with ischemic stroke in a Chinese population [41]. Elevated blood lipids is a risk factor for cardiovascular disease (CVD) [42]; however, the role of CYP2C19 gene variations in CVD remains to be investigated [41]. Hypercholesterolemia, hypertriglyceridemia, and hypertension are risk factor for atherosclerosis [42]. Atherosclerosis is an important cause of CAD [43]. This CYP2C family member catalyzes the metabolism of the arachidonic acid to endothelium-derived hyperpolarizing factor (EDHF), which causes blood vessel relaxation, including the coronary arteries [44]. The defective production of EDHF (due to CYP2C19 gene variants) can cause renal dysfunction and CVD [44]. In addition, the reactions catalyzed by CYP2C generate ROS in coronary arteries [44]. ROS suppress the relaxation of blood vessels regulated by nitric oxide [44].

Our results showed that the A allele of miR-423 rs6505162 C>A SNV is associated with CAD (Table 4 and Table 7). We also found that there was a significant difference in the miR-423 rs6505162 C>A genotype distribution between cases with normal lipid profile and cases with dyslipidemia (Figure 2C). Our results showed that there was a significant difference in miR-423 rs6505162 C>A genotype distribution between cases with normal and reduced HDL-C (Figure 2C). The miR-423 rs6505162 C>A genotype distribution was also significantly different between cases with normal blood pressure and cases with hypertension (Figure 2C). Elevated blood cholesterol and triglyceride are important risk factors for atherosclerosis [45]. Reduced HDL and hypertension also represent risk factors for atherosclerotic CVD [46,47]. This result confirmed the results of a previous study which showed that miR-423 rs6505162 C>A is associated with CAD in an Indian population [48]. The rs6505162 A allele enhances the expression of both mature miR-423 sequences (3p and 5p) [49]. Our result is consistent with a study, which reported that miR423-5p is increased in patients with heart failure (HF), and that elevated miR423-5p plasma levels are suggested as a biomarker for HF [50]. Our result is also in agreement with a study that reported that miR-423-5p downregulation inhibits gluconeogenesis and ameliorates insulin resistance (IR), increasing plasma fatty acid and glucose levels in obese diabetic mice [51]. In contrast, miR-423-5p overexpression in liver tissue enhances gluconeogenesis and increases blood sugar and the deposition of lipids in healthy mice [51]. Moreover, phosphoinositide 3-kinase (PI3K) family genes such as PIK3R3 are regulated by miR-423-5p. The increased expression of mir-423-5p by miR-423 rs6505162 C>A may lead to the accumulation of lipids and the inhibition of the insulin signaling pathway, resulting in insulin resistance [21]. Furthermore, miR-423-5p targets insulin receptor substrates 2; elevated expression of mir-423-5p by miR-423 rs6505162 C>A would therefore also result in insulin resistance [21]. The IR or impaired insulin action is the result of the defective metabolism of glucose in the hepatocytes, myocytes, and adipocytes [52]. IR is regarded as an initiator of cardiometabolic disorders such as type 2 diabetes and CVD [52]. In addition, miR-423-5p regulates TNFAIP3 Interacting Protein 2 (TNIP2), which negatively regulates NF-κB, suggesting the involvement of miR-423-5p in the axis of TNIP2-NF-κB [20]. NF-κB is a primary transcription factor which plays an important role in the induction of atherosclerosis by regulating the immune response and expression of genes involved in inflammation [53]. It has been proposed that miR-423-5p is implicated in CAD from an inflammatory perspective [20].

Limitations in our study include the limited sample size and the fact that it was a cross-sectional study. Large-scale longitudinal case control and protein functional studies are recommended to verify these findings.

## 5. Conclusions

In summary, the current study examined the association of TNF-α rs1800629 G>A, CYP2C19*17 (rs12248560) C>T, and miR-423 rs6505162 C>A with the induction of CAD in the Saudi population. Our result indicated that the GA and A allele of TNF-α—308 G>A (rs1800629) and the CYP2C19*17 (rs12248560) CT and T allele are linked to CAD. In addition, our results showed that the A allele of miR-423 rs6505162 C>A SNV is associated with CAD development in the Saudi population. We also observed that TNF-α plasma levels were significantly elevated in CAD cases compared to healthy controls. Future protein functional and large-scale case control investigations are required to confirm these results. After being confirmed, our results could be utilized in genetic testing to identify populations susceptible to CAD.

## Figures and Tables

**Table 1:** ARMS primers of *TNF-α rs1800629 G>A*, miR-423 rs6505162 C>A and CYP2C19*17-rs12248560 *C>T* gene polymorphism.

Amplification-Refractory Mutation System Primers of *TNF-α rs1800629 G>A* Genotyping					Ref
TNF-α FI	(A allele)	5′-AGTTGGGGACACGCAAGCATGAAGGATA-3′	60 °C	154 bp	[22]
TNF-α RI	(G allele)	5′-TAGGACCCTGGAGGCTAGACCCCGTACC-3′		224 bp	
TNF-α Fo		5′-ACCCAAACACAGGCCTCAGGACTCAACA-3′		323 bp	
TNF-α Ro		5′-TGGAGGCAATAGCTTTTGAGGGGCAGGA-3′			
**Amplification-Refractory Mutation System Primers of miR-423 rs6505162 C>A Genotyping**					
miR-423 FI	(A allele)	5′-TGAGGCCCCTCAGTCTTGCTTCCCAA-3′	62 °C	228 bp	[23]
miR-423 RI	(C allele)	5′-CAAGCGGGGAGAAACTCAAGCGCGAGG-3′		160 bp	
miR-423 Fo		5′-TTTTCCCGGATGGAAGCCCGAAGTTTGA-3′		336 bp	
miR-423 Ro		5′-TTTTGCGGCAACGTATACCCCAATTTCC-3′			
**Amplification-Refractory Mutation System Primers of** CYP2C19*17- **rs12248560 ** ***C>T *** **Genotyping**					
CYP2C19*17 FI	T allele	5′-TTTTTCAAATTTGTGTCTTCTGTTCTCAAATT-3′	56 °C	227 bp	[24]
CYP2C19*17 RI	C allele	5′-GCGCATTATCTCTTACATCAGAGCTG-3′		292 bp	
CYP2C19*17 Fo		5′-GAGATCAGCTCTTCCTTCAGTTACAC-3′		462 bp	
CYP2C19*17 Ro		5′-CACCTTTACCATTTAACCCCCTAAAAA-3′			

Abbreviations: FI, forward primer; FO, forward outer primer; RI, reverse inner primer; RO, reverse outer primer.

**Table 2:** Demographic features of CAD patients and healthy controls.

	*n* = 104	%	*n* = 114	%
Male	80	76.92	74	64.91
Female	24	23.07	40	35.08
Age < 50	69	66.34	64	56.14
Age > 50	35	33.65	50	43.85
**Lipid Biomarkers**				
HDL ≤ 40 (mg/dL)	49	47.11		
HDL > 40 (mg/dL)	55	52.88		
Cholesterol ≤ 200 (mg/dL)	45	43.26		
Cholesterol > 200 (mg/dL)	59	56.73		
TGL ≤ 150 (mg/dL)	43	41.34		
TGL > 150 (mg/dL)	61	58.65		
LDL ≤ 100 (mg/dL)	41	39.42		
LDL > 100 (mg/dL)	63	60.57		
**Clinical Complications**				
Hypertension	70	67.30		
No hypertension	34	32.70		
T2D	39	37.5		
No T2D	65	62.5		
Myocardial infarction	24	23.07		
No Myocardial infarction	80	76.92		
Obesity	14	13.46		
No Obesity	90	86.53		

*p*-values > 0.05 were considered significant.

**Table 3:** Age and blood biochemistry of CAD patients and healthy controls.

Group		Mean ± Std.	*p*-Value
Age	Cases	35.33 ± 14.31	0.090
	Control	39.36 ± 12.20	
FBG (mmol/L)	Cases	7.66 ± 2.34	<0.05
	Controls	6.69 ± 2.10	
**Lipid profile**			
Cholesterol	Cases	129.22 ± 10.11	<0.00012
	Controls	110.31 ± 8.01	
VLDL	Cases	40.09 ± 7.01	<0.00013
	Controls	27.98 ± 5.58	
Triglyceride	Cases	151.16 ± 15.01	<0.00012
	Controls	125.70 ± 8.99	
HDL	Cases	33.90 ± 8.81	<0.00013
	Controls	28.27 ± 5.10	
LDL	Cases	185.70 ± 33.37	<0.00012
	Controls	118.83 ± 33.37	

*p*-values > 0.05 were considered significant.

**Table 4:** Distribution and association of the TNF-α rs1800629 G>A, CYP2C19*17—rs12248560 C>T, and miR-423 rs6505162 C>A genotypes and alleles between CAD patients and controls.

Potential Association of the TNF-α rs1800629 G>A Genotypes with CAD									
Subjects	*n* = 208	GG	GA	AA	Df	X2	G	A	*p*-Value
CAD	104	48 (46.15%)	44 (42.30%)	12 (11.53%)	2	10.7	0.67	0.33	0.006
Controls	104	70 (67.30%)	29 (27.88%)	5 (4.80%)			0.81	0.19	
Potential Association of the CYP2C19*17—rs12248560 C>T Genotypes with CAD									
**Subjects**	***n *** **= 209**	**CC**	**CT**	**TT**	**Df**	**X2**	**C**	**T**	***p*-Value**
Cases	105	55 (52.88%)	39 (37.5%)	11 (10.47%)	2	7.98	0.71	0.29	0.018
Controls	104	73 (70.19%)	27 (25.96%)	04 (3.84%)			0.84	0.16	
**Potential Association of the miR-423 rs6505162 C>A Genotypes with CAD**									
**Subjects**	***n *** **= 218**	**CC**	**CA**	**AA**	**Df**	**X2**	**C**	**A**	***p*-Value**
Cases	104	23 (22.11%)	48 (46.15%)	33 (31.73%)	2	6.0	0.46	0.54	0.049
Controls	114	32 (28.07%)	62 (54.38%)	20 (17.54%)			0.55	0.45	

*p*-values > 0.05 were considered significant.

**Table 5:** Logistic regression to estimate the association of TNF-α rs1800629 G>A gene polymorphism with the risk of CAD susceptibility.

Genotypes	Healthy Controls (*n* = 104)	CAD Cases (*n* = 104)	Odd Ratio OR (95% CI)	Risk Ratio RR (95% CI)	*p*-Value
**Codominant model**					
TNF-α—GG	70	48	1 (ref.)	1 (ref.)	
TNF-α—GA	29	44	2.21 (1.2197 to 4.0138)	1.49 (1.0847 to 2.0557)	0.009
TNF-α—AA	05	12	3.12 (1.1580 to 10.578)	2.1 (0.9514 to 4.2761)	0.026
**Dominant model**					
TNF-α—GG	70	48	1 (ref.)	1 (ref.)	
TNF-α (GA + AA)	34	56	2.40 (1.3685 to 4.2159)	1.57 (1.1583 to 2.1289)	0.002
**Recessive model**					
TNF-α—(GA + GG)	99	92	1 (ref.)	1 (ref.)	
TNF-α—AA	05	12	2.58 (0.8760 to 7.6142)	1.76 (0.8760 to 7.6142)	0.085
Allele					
TNF-α—G	169	140	1 (ref.)	1 (ref.)	
TNF-α—A	39	68	2.10 (1.3381 to 3.3107)	1.50 (1.1455 to 1.9656)	0.0013
**Over dominant model**					
TNF-α—(GG + AA)	75	60	1 (ref.)	1 (ref.)	
TNF-α (GA)	29	44	1.89 (1.063 to 3.383)	1.30 (1.0152 to 1.9265)	0.030

*p*-values > 0.05 were considered significant.

**Table 6:** Logistic regression to estimate the association of CYP2C19*17 (rs12248560) C>T genotypes with CAD susceptibility.

Genotypes	Healthy Controls	CAD Cases	OR (95% CI)	Risk Ratio (RR)	*p*-Value
	(*n* = 104)	(*n* = 105)			
**Codominant model**					
CYP2C19*17—CC	73	55	1 (ref.)	1 (ref.)	
CYP2C19*17—CT	27	39	1.91 (1.0493 to 3.5028)	1.39 (1.0056 to 1.9326)	0.030
CYP2C19*17—TT	04	11	3.65 (1.1030 to 12.078)	2.13 (0.9117 to 5.0167)	0.034
**Dominant model**					
CYP2C19*17—CC	73	55	1 (ref.)	1 (ref.)	
CYP2C19*17—(CT + TT)	31	50	2.14 (1.2124 to 3.7799)	1.49 (1.2124 to 3.7799)	0.0087
**Recessive model**					
CYP2C19*17—(CC + CT)	100	94	1 (ref.)	1 (ref.)	
CYP2C19*17—TT	04	11	2.92 (0.9003 to 9.5067)	1.93 (0.8260 to 4.5236)	0.074
**Allele**					
CYP2C19*17—C	173	149	1 (ref.)	1 (ref.)	
CYP2C19*17—T	35	61	2.02 (1.2650 to 3.2371)	1.47 (1.1106 to 1.9554)	0.003
**Over dominant model**					
CYP2C19*17—CC + TT	77	94	1 (ref.)	1 (ref.)	
CYP2C19*17—CT	27	66	2.0 (1.1673 to 3.4349)	1.55 (1.0839 to 2.2194)	0.0117

*p*-values > 0.05 were considered significant.

**Table 7:** Logistic regression to estimate the association of miR-423 rs6505162 C>A genotypes with CAD susceptibility.

Genotypes	Healthy Controls (*n* = 114)	CAD Cases (*n* = 104)	OR (95% CI)	Risk Ratio (RR)	*p*-Value
**Codominant inheritance model**					
MicroR-423—CC	32	23	1 (ref.)	1 (ref.)	
MicroR-423—CA	62	48	1.07 (0.5595 to 2.0737)	1.03 (0.7818 to 1.3630)	0.82
MicroR-423—AA	20	33	2.29 (1.0611 to 4.9667)	1.54 (1.0611 to 4.9667)	0.034
**Dominant inheritance model**					
MicroR-423—CC	32	23	1 (ref.)	1 (ref.)	
MicroR-423—(CA + AA)	82	81	1.37 (0.7411 to 2.5485)	1.15 (0.8819 to 1.5167)	0.31
**Recessive inheritance model**					
MicroR-423—(CC + CA)	94	71	1 (ref.)	1 (ref.)	
MicroR-423—AA	20	33	2.18 (1.1574 to 4.1230)	1.50 (1.0424 to 2.1865)	0.029
Allele					
MicroR-423—C	126	94	1 (ref.)	1 (ref.)	
MicroR-423—A	102	114	1.49 (1.0268 to 2.1858)	1.21 (1.0116 to 1.4541)	0.036
**Over dominant Inheritance model**					
MicroR-423—CC + AA	52	56	1 (ref.)	1 (ref.)	
MicroR-423—CA	62	48	0.71 (0.4217 to 1.2255)	0.85 (0.6616 to 1.1031)	0.225

The *p*-values > 0.05 were considered significant.

## Data Availability

All the data associated with the current study has been presented in this manuscript.
